# Population Genetics of the Medicinal Leech *Whitmania pigra* Based on Mitochondrial Genes

**DOI:** 10.3390/genes17101278

**Published:** 2026-10-14

**Authors:** Yanping Pan, Pinpin Zhou, Yiyong Luo, Ming Yang, Huanhuan Chen, Zichao Liu, Fang Zhao, Gonghua Lin, Lizhou Tang

**Affiliations:** 1 Key Laboratory of Biodiversity Conservation and Bioresource Utilization of Jiangxi Province, College of Life Sciences, Jiangxi Normal University, Nanchang 330022, China; 15581461344@163.com (Y.P.); zhou166833@outlook.com (P.Z.); yyongl-168@163.com (Y.L.); 2 Cancer Center, Laboratory of Oncogene, State Key Laboratory of Biotherapy, West China Hospital, Sichuan University, Chengdu 610041, China; qjyangming@163.com; 3 College of Biology and Food Engineering, Qujing Normal University, Qujing 655011, China; chhuanhuan@163.com; 4 Yunnan International Joint Laboratory with South and Southeast Asia for the Integrated Development of Animal-Derived Anti-Thrombosis Chinese Medicine, Qujing Normal University, Qujing 655011, China; 5 Yunnan Key Laboratory of Leech Bioresources and Industrialization, School of Agriculture and Life Sciences, Kunming University, Kunming 650214, China; abclzc@aliyun.com; 6 School of Life Sciences, Key Laboratory of Jiangxi Province for Functional Biology and Pollution Control in Red Soil Regions, Jinggangshan University, Ji’an 343009, China; zf_lgh@163.com

**Keywords:** *Whitmania pigra*, mitochondrial protein-coding genes, genetic analysis, demographic history

## Abstract

**Background**: Leeches possess significant medicinal value as antithrombotic agents, and elucidating their population genetic background is of great importance for the conservation of medicinal germplasm resources and the development of pharmaceutical applications. **Methods**: In this study, mitochondrial genes (*COI*, *Cytb*, and *ND1*) were used as molecular markers to systematically investigate the population genetic characteristics of wild *Whitmania pigra* from 13 regions across China. A total of 137 individuals were sequenced, yielding a concatenated alignment of 3554 bp with 441 variable sites. Haplotype networks and phylogenetic trees were constructed based on the concatenated sequences, alongside BAPS clustering, neutrality tests, and Bayesian Skyline Plot (BSP) analyses. **Results**: Genetic diversity analyses revealed that the species-level concatenated dataset and most populations exhibited high haplotype diversity (Hd > 0.5) and low nucleotide diversity (Pi < 0.02), with JXJA as a notable exception (concatenated Hd = 0.200). Neutrality tests (Fu’s Fs = −6.002, *p* = 0.001) supported a scenario of historical bottlenecks followed by rapid demographic expansion, whereas the reduced diversity in JXJA may reflect a local bottleneck or founder effect. Haplotype networks, phylogenetic trees, and BAPS clustering consistently divided the 13 populations into three significantly differentiated groups: northeastern (HLJ), southern, and northern-central. The HLJ population exhibited extremely high genetic differentiation (F_ST_ = 0.902–0.977), suggesting it may represent a highly differentiated mitochondrial lineage and a candidate Evolutionarily Significant Unit (ESU). The BSP analysis reconstructed four phases of demographic history, broadly corresponding to climatic fluctuations since the Late Pleistocene. **Conclusions**: Overall, this study reveals high haplotype diversity and low nucleotide diversity in *W. pigra*, with JXJA representing a distinct local bottleneck case. The populations are strongly structured into three lineages, with the HLJ population representing a highly differentiated mitochondrial lineage and a candidate ESU. Demographic dynamics appear broadly associated with Late Pleistocene climatic fluctuations, though the timing remains uncertain due to the substitution rate. These findings provide a basis for *W. pigra* conservation, and future studies incorporating nuclear markers are warranted.

## 1. Introduction

Leeches are annelids belonging to the class Hirudinea [1], a group of invertebrates widely distributed in diverse freshwater environments worldwide (e.g., ponds, rivers, and marshes), with a few species also inhabiting marine or moist terrestrial ecosystems. Globally, about 680 leech species have been recorded, and they play important ecological roles [2], acting as intermediate predators or parasites in material cycling and energy flow, while also possessing remarkable medical value. Leech saliva contains multiple potent bioactive components, among which hirudin is the most prominent [3,4]. This substance was first extracted by Haycraft in 1884 [5] and later named by Jacoby in 1904 [5]. As the most potent natural specific thrombin inhibitor known to date [6], hirudin can efficiently and precisely inhibit thrombin activity [7], preventing the conversion of fibrinogen to fibrin [8], thus exhibiting strong anticoagulant effects and being regarded as a representative natural anticoagulant [9]. Beyond its anticoagulant function, hirudin has multiple pharmacological potentials. Liu et al. [10] found that it exerts protective effects against diabetic nephropathy, nephrotic syndrome, and renal interstitial fibrosis in chronic kidney disease. Moreover, other leech extracts have been used for topical treatment of knee osteoarthritis, showing anti-inflammatory regulatory functions [11,12], and have demonstrated certain capacities to inhibit cancer cell proliferation and metastasis [13]. They also show application prospects in anti-fibrosis and regulation of metabolic disorders [14], treatment of herpes zoster [15], and promotion of skin wound healing [16]. In China, more than 90 leech species have been recorded, exhibiting a distinct south-to-north distribution gradient. They are mainly concentrated in the middle and lower reaches of the Yangtze River, Southwest China, and South China, where water systems are well-developed and climates are humid. In contrast, Northern populations are primarily found in the wetlands of Northeast China and localized areas of the Yellow River basin [17]. Among them, medicinal leeches have been extensively studied, such as *Whitmania pigra* and *Hirudo nipponia*, which are endemic or common species with significant economic and medicinal value [18,19,20,21].

*W. pigra* is one of the most widely distributed and medicinally valuable leech species in China, commonly found in freshwater bodies across various provinces, as well as in Russia, Japan, and other countries [1,22]. It has a moderate body size, typical suckers, well-developed jaws and teeth, and a complete digestive system [23,24,25]. It feeds on the body fluids of mollusks and prefers warm, shallow waters with stone and algae attachment [26]. More importantly, this species has been included in the Chinese Pharmacopoeia [27] and is currently the most widely used medicinal leech variety in clinical practice in China, with advantages such as accessible resources, strong ecological adaptability, and clear efficacy. It has thus become the core subject of research and utilization of leech medicinal materials, holding irreplaceable scientific and industrial importance [28,29,30,31,32].

Existing basic biological studies on this species are mostly focused on macroscopic aspects such as taxonomy, feeding habits, distribution, and behavioral traits, and remain relatively fragmented [33]. Taxonomically, the family placement of the genus Whitmania and the delimitation of closely related species are still controversial; traditional morphological classification cannot accurately distinguish sibling species, and the taxonomic system is not yet unified [34,35]. Feeding studies are mostly based on artificial breeding conditions, clarifying the effects of environmental factors on feeding and growth [36,37,38], but systematic investigations of long-term feeding adaptation mechanisms in natural populations are lacking. Distribution and behavioral studies have only provided a preliminary outline of macro-distribution patterns and basic activity rhythms; limited molecular marker work has been restricted to local populations, failing to elucidate population differentiation and behavioral adaptive evolution on a broader scale [39,40,41], and mechanistic studies at the population level are clearly insufficient. Conducting population genetics research holds significant theoretical and applied value. In application, it can clarify taxonomic and germplasm differences, providing a basis for medicinal material identification, quality standards, and medication safety; assess wild diversity to support conservation, guide selective breeding, and alleviate germplasm bottlenecks. In theory, it can explain population differentiation and adaptive mechanisms, analyze the genetic regulation of medicinal traits, and fill research gaps. In summary, this study is key to supplementing fundamental knowledge and promoting resource conservation and industrial development.

Mitochondrial DNA (mtDNA), as a molecular marker, is widely used in population genetics and phylogeography due to its unique genetic characteristics: strict maternal inheritance with no recombination during transmission, providing a clear and traceable genetic lineage for tracking maternal ancestry and population history [42]; a relatively high nucleotide substitution rate, accumulating abundant variable sites within species and among closely related species [43], sensitively reflecting population genetic diversity and differentiation patterns; high copy number and relatively conserved structure, allowing easy design of universal primers and high PCR amplification success rates, enabling stable information retrieval even from degraded samples [44,45]. The *COI* gene has been recognized as the standard DNA barcode for animals, and the *Cytb* gene is also a classic marker in population genetics studies of vertebrates and invertebrates [46,47,48], both serving dual functions of species identification and genetic diversity analysis, achieving “one marker for multiple uses”. Müller et al. [49] pointed out that mitochondrial protein-coding genes (including *COI*, *Cytb*, etc.) perform well in phylogenetic reconstruction of non-primate invertebrates; Tong et al. [50] also emphasized the potential application of such genes in exploring bioactive components in the *W. pigra* genome. However, mtDNA markers also have limitations, such as an effective population size only one-quarter that of nuclear genes, maternal inheritance failing to reflect male-mediated gene flow, and potential interference from nuclear mitochondrial pseudogenes (NUMTs) [51]. In practical applications, marker strategies should be carefully chosen and, when necessary, combined with nuclear markers for cross-validation [52].

Previous molecular population genetics studies on *W. pigra*, although using various markers, still have notable limitations. First, early studies relied on dominant markers with poor reproducibility and limited information content. Liu et al. [53] used ISSR and SRAP markers to analyze genetic diversity and detected a high proportion of polymorphic loci, but these dominant markers cannot distinguish homozygous from heterozygous states, making accurate estimation of genetic parameters difficult; amplification results are easily affected by PCR conditions, resulting in poor experimental reproducibility and difficult integration of data across laboratories. Second, geographic coverage was narrow, failing to reveal a national-scale differentiation pattern. Jiang et al. [54] used RAPD markers to analyze five populations from Xinghua (Jiangsu), Binxian (Heilongjiang), Dawu (Hubei), etc., and found significant regional differences, but sampling was still limited to parts of East and North China, without covering major southern distribution areas such as Sichuan, Hunan, Jiangxi, and Anhui. The generality of conclusions was constrained, insufficient for supporting national resource conservation planning. To date, no study has systematically depicted the genome-wide genetic structure of *W. pigra* across China. Third, single-gene marker strategies provide limited phylogenetic information. Yue et al. [55] used only the *Cytb* single gene to analyze three wild populations; although they detected 47 haplotypes with high haplotype diversity (Hd = 0.9781), the limited variable sites of a single gene cannot deeply reveal complex evolutionary relationships and differentiation histories. Hao et al. [56] expanded the sample to five populations and 146 individuals using *COI* and *Cytb* dual-gene markers, but were still restricted to two mitochondrial genes, lacking a more comprehensive genomic perspective.

This study adopted a combined three-gene (*COI*, *Cytb*, *ND1*) marker strategy, providing richer variable sites and phylogenetic information compared to single- or double-gene studies. Sampling covered 13 geographic populations across China with 137 individuals, involving Heilongjiang, Shandong, Anhui, Jiangsu, Hubei, Hunan, Jiangxi, Henan, Sichuan, Tianjin, and other major distribution areas, with substantially expanded geographic coverage and sample size relative to previous work. Multiple analytical methods were comprehensively applied to systematically dissect the genetic structure, differentiation patterns, and demographic history of the national populations. Collectively, this study achieves a systematic advancement over existing studies in terms of marker number, geographic coverage, sample size, and analytical approaches. The results will provide a solid scientific basis for formulating germplasm resource conservation strategies, defining Evolutionarily Significant Units (ESUs), and guiding selective breeding of *W. pigra*.

## 2. Materials and Methods

### 2.1. Sample Collection and Sequencing

**Figure 1:**
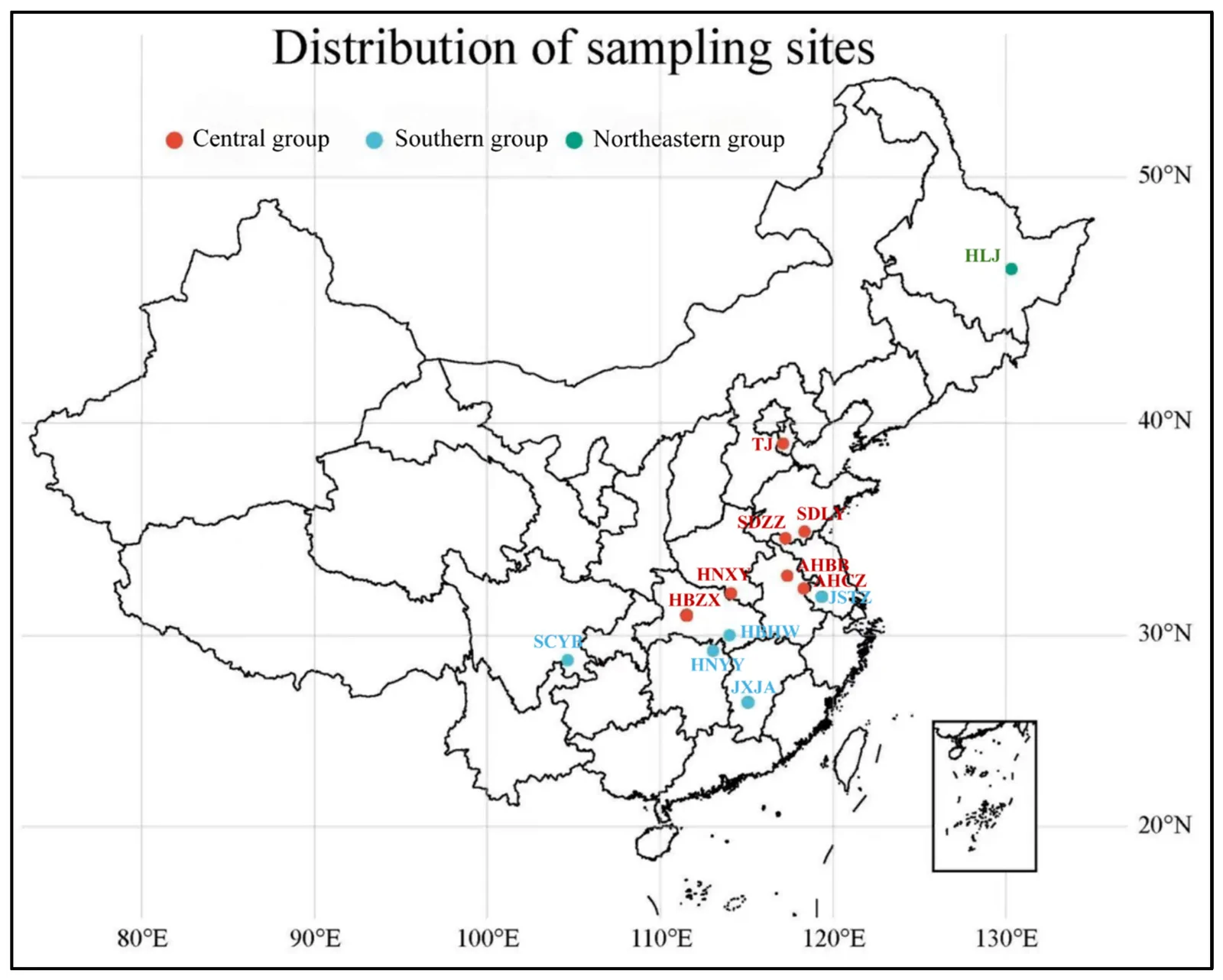
Distribution map of sampling points for *W. pigra*.

A total of 137 wild *W. pigra* individuals were collected from 13 localities across China (Figure 1, Table 1). 10–12 individuals were collected from each population. Anterior tissue was excised from each individual, and total genomic DNA was extracted using the DNeasy Blood and Tissue Kit (Qiagen, Hilden, Germany). Qualified DNA samples were used to construct libraries with ~350 bp insert fragments using Illumina-compatible library preparation kits, and 150 bp paired-end whole-genome resequencing was performed on the BGISEQ sequencing platform. Raw data were quality-controlled using FASTP v0.20.0 [57] to obtain clean reads for each sample.

### 2.2. Extraction of Mitochondrial Gene Sequences

The clean reads obtained from resequencing were assembled de novo using MEGAHIT v1.2.9 [58] to generate contig sequence files. This study focused on three mitochondrial protein-coding genes (*COI*, *Cytb*, and *ND1*) for population genetic analyses of *W. pigra*. Using the published mitochondrial genome of *W. pigra* as reference, the *COI*, *Cytb*, and *ND1* sequences were used as bait sequences to search for homologous sequences in the assembled contigs via BLAST v2.13.0 [59]. The retrieved homologous sequences were aligned using MEGA v11.0.13 [60], and the homologous regions were accurately extracted. To exclude nuclear mitochondrial pseudogenes (NUMTs), the retrieved contigs were validated against the published *W. pigra* mitochondrial genome by requiring a minimum BLAST identity of 98% and coverage of 90%, along with intact open reading frames lacking premature stop codons or frameshifts, and consistent read coverage across the entire gene region. The average sequencing depth across the mitochondrial genome was >100×, which is substantially higher than that of the nuclear genome and sufficient for reliable assembly and variant calling.

### 2.3. Genetic Diversity Analysis

The coding region sequences of the three target mitochondrial protein-coding genes were integrated into separate FASTA files (strictly restricted to CDS regions). Subsequently, multiple sequence alignment was performed using the “Align by MUSCLE” function in MEGA v11.0.13. Based on the alignment results, DnaSP v6 [61] was used to calculate genetic diversity parameters at the population level for each gene: number of variable sites (S), number of haplotypes (Hn), haplotype diversity (Hd), and nucleotide diversity (Pi). The three genes were concatenated, and the same parameters were calculated for the combined dataset across all populations. To evaluate genetic variation within each geographic population, DnaSP v6 was also used to calculate the above parameters for each individual gene and the concatenated genes within each population.

### 2.4. Haplotype Networks and Phylogenetic Analysis

Based on the sequence datasets of the three genes (*COI*, *Cytb*, and *ND1*), haplotype network construction and phylogenetic analyses were performed separately for each gene. First, haplotypes were extracted from the alignment results of each gene using DnaSP v6. Then, HapSolutely v0.2.2 [62] was used to construct gene-specific haplotype networks under the Fitch model. Subsequently, homologous sequences of the closely related species *Whitmania laevis* (GenBank accession: NC_023926.1) were downloaded as Outgroup. MEGA11 was used to construct maximum-likelihood (ML) phylogenetic trees for haplotypes of each gene and for the combined haplotypes, with the optimal nucleotide substitution model selected using MEGA11’s built-in model selection tool. All analyses were performed with 1000 bootstrap replicates to assess node support. All analyses were performed with 1000 bootstrap replicates to assess node support. The resulting tree files were visualized and optimized using FigTree v1.4.4 (http://tree.bio.ed.ac.uk/software/Figtree/, accessed on 20 August 2025). The same methods were applied to the concatenated gene dataset for haplotype network construction and phylogenetic analysis.

### 2.5. Analysis of Population Genetic Structure

Bayesian clustering analysis was performed using BAPS v6.0 [63] for the concatenated mitochondrial genes. The number of clusters (K) was explored from K = 2 to K = 7, with each K value repeated 5 times to ensure MCMC convergence. Four input files were prepared: sequence data, population names, indices of the first individual per population, and a linkage map. The “Clustering with linked loci” module was used for population mixture analysis. Genetic structure plots were generated with BAPS. Pairwise F_ST_ values between populations were calculated using Arlequin v3.5 [64]. Permutation tests were performed with 1000 replicates, and the significance level (α) was set at 0.05.

### 2.6. Analysis of Population Historical Dynamics

The Bayesian Skyline Plot (BSP) model implemented in BEAST v1.10.4 [65] was used to reconstruct the historical effective population size (Ne) changes of *W. pigra*. Because different genes have different mutation rates, making time unification difficult, the three genes were concatenated for analysis to better explore the population demographic history. The optimal nucleotide substitution model was selected using jModelTest v2.1.10 [66]. Analysis parameters were configured in BEAUti v1.10.4 [66] using a partitioned model with estimated base frequencies, an uncorrelated lognormal relaxed clock, and a Bayesian Skyline Plot tree prior with 10 stepwise control points. To address the uncertainty of applying an insect mitochondrial substitution rate to annelids, the clock rate was assigned a lognormal prior with a mean of 0.0135 (1.35% per site per million years) and a standard deviation of 0.005, rather than being fixed. The MCMC chain was run for 10,000,000 generations, sampling every 1000 generations, with the first 10% discarded as burn-in. Two independent runs were performed and combined in Tracer v1.7 to assess convergence, with effective sample size (ESS) values exceeding 200 for the vast majority of parameters. In addition to the BSP analysis, Tajima’s D and Fu’s Fs neutrality tests, as well as mismatch distribution analysis, were performed using DnaSP v6 to further test the demographic expansion scenario. Harpending’s raggedness index was calculated via coalescent simulations (1000 replicates) under a population growth model to evaluate the goodness-of-fit of the observed mismatch distribution to the expected model. Given the significant population genetic structure, all analyses were conducted for the concatenated dataset of all individuals (*n* = 137) and separately for the three identified lineages (Southern, Central, and Northeastern groups).

## 3. Results

### 3.1. Variations in Gene Sequence

We obtained sequences of the target genes *COI*, *Cytb*, and *ND1* from 137 individuals across 13 populations. Among them, *COI* had the longest length (1534 bp), and *ND1* the shortest (874 bp). Alignment revealed extensive genetic variation, with a total of 441 variable sites identified, ranging from 104 to 182 variable sites and 53 to 78 haplotypes per gene. *COI* had the highest number of variable sites and haplotypes, while *ND1* had the lowest, indicating that sequence length substantially affects variation. For nucleotide diversity, *Cytb* ranked highest, followed by *COI*, while the shortest gene *ND1* remained the lowest (Table 2).

### 3.2. Genetic Diversity Within a Gene Population

Using DnaSP v6, genetic diversity parameters were calculated for the 13 geographic populations of *W. pigra* based on *COI*, *Cytb*, and *ND1* sequences (Table 3), including the number of variable sites (S), haplotype diversity (Hd), and nucleotide diversity (Pi), with combined information after concatenation.

Among the individual genes, *COI* exhibited the highest level of genetic variation, with 182 variable sites in total. JSTZ and HBWH had the highest number of variable sites (41 each), while JXJA had none (S = 0). Haplotype diversity (Hd) was highest in JSTZ (1.000) and zero in JXJA, indicating no variation detected in the *COI* gene for that population. Nucleotide diversity (Pi) was generally moderate to high, consistent with the distribution of variable sites. For *Cytb*, total variable sites were 155, with TJ having the most (38). The highest Hd was in JSTZ (0.978) and the lowest in JXJA (0.200). Nucleotide diversity for this gene remained moderately high in most populations, suggesting a relatively high evolutionary rate. For *ND1*, total variable sites were 104, the lowest among the three, indicating stronger conservation. HBWH had the most polymorphic sites (18). Hd was highest in TJ (0.939) and still lowest in JXJA (0). Nucleotide diversity followed a similar pattern, further supporting the lower variability of *ND1*.

After concatenating the three genes, the total number of variable sites was 441 (Table 4), JSTZ still exhibited extremely high genetic diversity in the combined dataset (Hd = 1.000), highlighting its role as a reservoir of comprehensive genetic variation. highlighting its comprehensive accumulation of genetic variation. JXJA had a combined Hd of only 0.200, significantly lower than other populations, indicating a relatively homogeneous genetic background. Overall, different mitochondrial genes showed marked differences in genetic variation, with *COI* being the most variable and *ND1* the most conserved. JSTZ consistently showed high or the highest genetic diversity across all three genes and the combined dataset, suggesting it may be a genetic diversity center for *W. pigra* with important conservation significance. Conversely, JXJA consistently showed low diversity, possibly having experienced a genetic bottleneck or founder effect, and thus requires priority genetic conservation measures.

### 3.3. Haplotype Network Analysis

**Figure 2:**
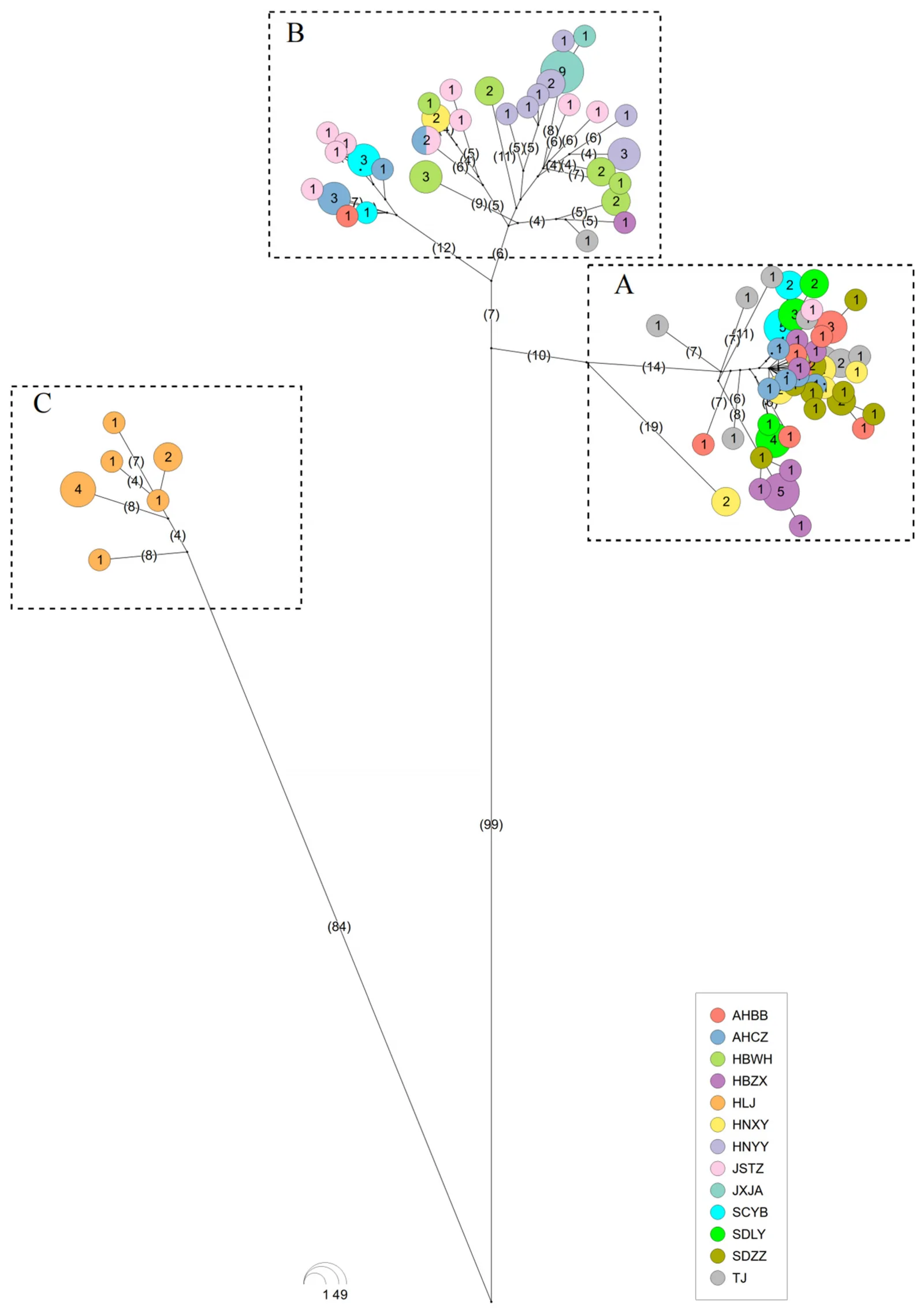
Haplotype network of *W. pigra* populations based on mitochondrial gene sequences.

The haplotype networks constructed based on the sequences of the three mitochondrial genes *COI*, *Cytb*, and *ND1* all exhibit similar topological structures, clearly divided into three major branches (A, B, and C), with a characteristic radial distribution from the central haplotype outward (Supplementary Figures S1–S3). Specifically, Branch A consists primarily of haplotypes derived from AHBB, HBZX, SDLY, SDZZ, and TJ, along with some haplotypes from AHCZ, HNXY, and SCYB; Branch B comprises mainly haplotypes from JXJA, HBWH, JSTZ, and HNYY, including some from AHCZ and SCYB; whereas Branch C demonstrates significant geographic specificity, with all its haplotypes originating exclusively from the HLJ population. The combined haplotype network for the three genes (Figure 2) yields identical results to those derived from individual genes.

### 3.4. Phylogenetic Analysis

**Figure 3:**
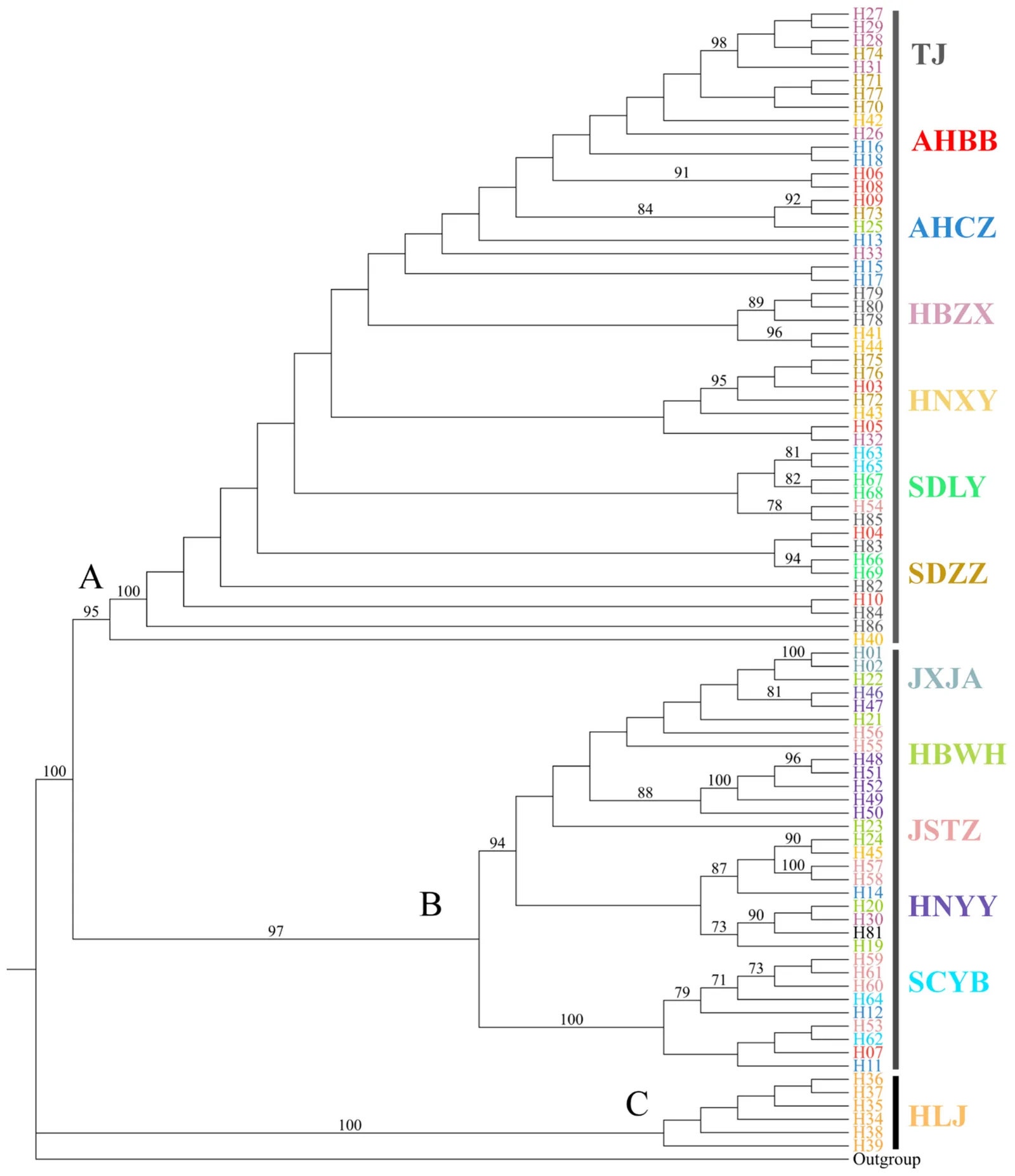
Phylogenetic relationship of haplotypes in the *W. pigra* population based on mitochondrial gene sequences.

Using the Polar Tree layout visualization feature in FigTree (Figures S4–S6). The haplotype-based phylogenetic trees for all three genes each exhibit three distinct branches (A, B, and C): Branch A primarily comprises the haplotypes AHBB, HBZX, SDLY, SDZZ, AHCZ, and TJ; Branch B consists mainly of JXJA, HBWH, HNYY, JSTZ, and SCYB; notably, Branch C contains only the haplotype HLJ. When the three genes were combined for phylogenetic analysis, the results aligned with those obtained from single-gene analyses (Figure 3), identifying three mitochondrial lineages (A, B, and C). This phylogenetic structure corresponds precisely to the findings from haplotype network analysis.

### 3.5. Genetic Structure and Genetic Differentiation

**Figure 4:**
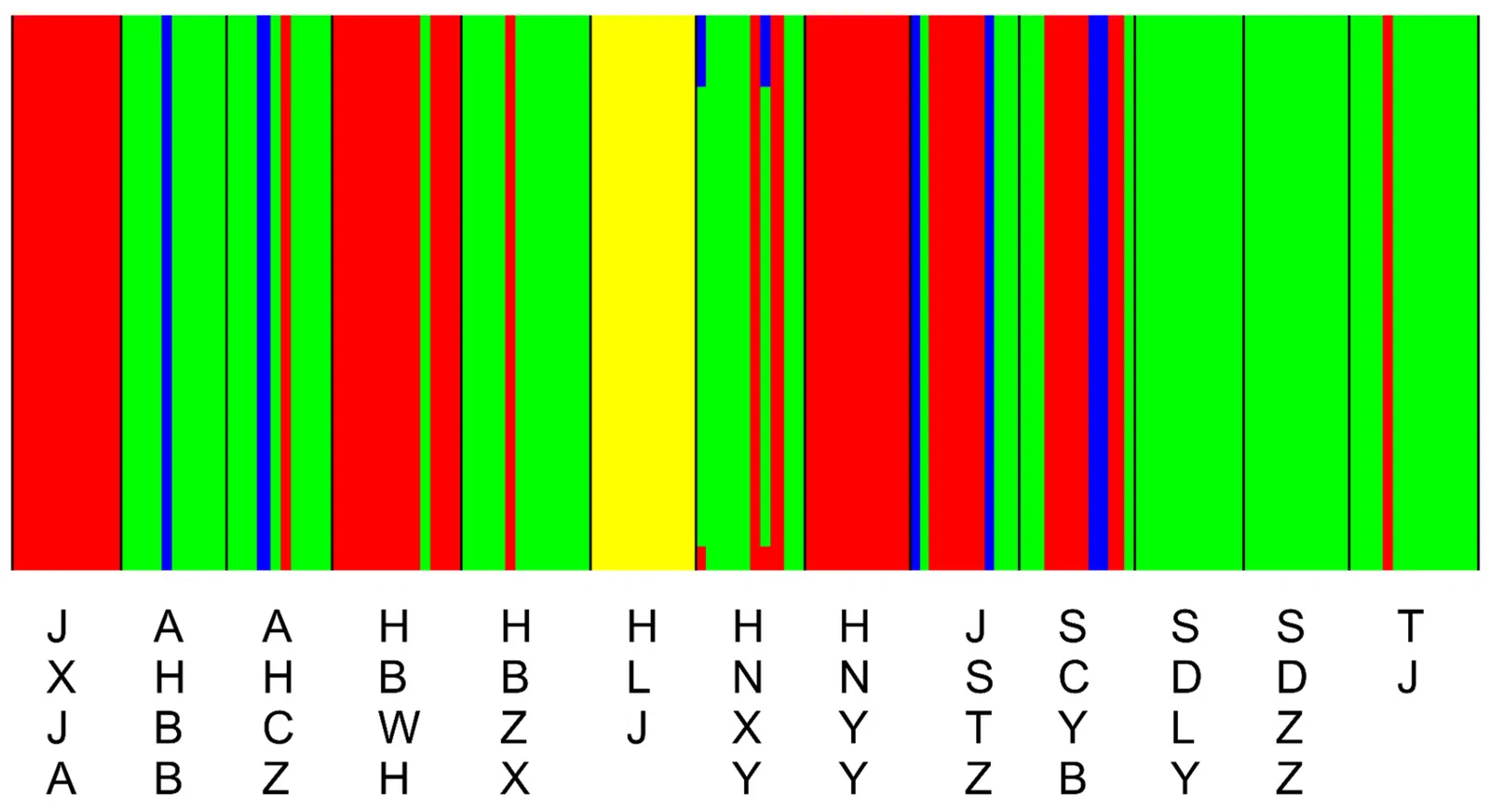
The population genetic structure patterns of *W. pigra* based on mitochondrial gene sequences under different clustering strategies (K = 2–7).

Bayesian clustering analysis using BAPS resolved the 137 individuals into three distinct genetic clusters (Figure 4). Notably, the HLJ population formed a separate cluster, exhibiting complete genetic isolation without evidence of recent admixture. This pattern strongly supported its recognition as a highly differentiated mitochondrial lineage and a candidate ESU. The southern group (JXJA, HNXY, HNYY, JSTZ, HBWH) was characterized predominantly by the red ancestral component, with JXJA consisting almost entirely of this component. In contrast, the northern-central group (AHBB, HBZX, SDLY, SDZZ, TJ) was dominated by the green component.

These clustering results were further corroborated by pairwise F_ST_ values (Table 5). The F_ST_ between HLJ and all other populations was exceptionally high (0.902–0.977, *p* < 0.001), confirming its pronounced genetic distinctiveness. Moreover, F_ST_ values between the southern and northern-central groups generally exceeded 0.5, indicating substantial genetic differentiation. These findings are highly congruent with the haplotype network and phylogenetic reconstructions, providing multiple lines of evidence for the observed structure. In summary, the population genetic structure of Chinese *W. pigra* comprises three well-defined evolutionary units: a genetically isolated northeastern group (HLJ), a southern group, and a northern-central group. This phylogeographic pattern suggests that historical geographic isolation, such as glacial refugia or riverine barriers, may have played a key role in shaping the species’ evolutionary history. The HLJ group, being the most genetically distinct, should be considered a highly differentiated mitochondrial lineage and a candidate ESU deserving priority for conservation.

### 3.6. Population Demography and Neutrality Tests

**Figure 5:**
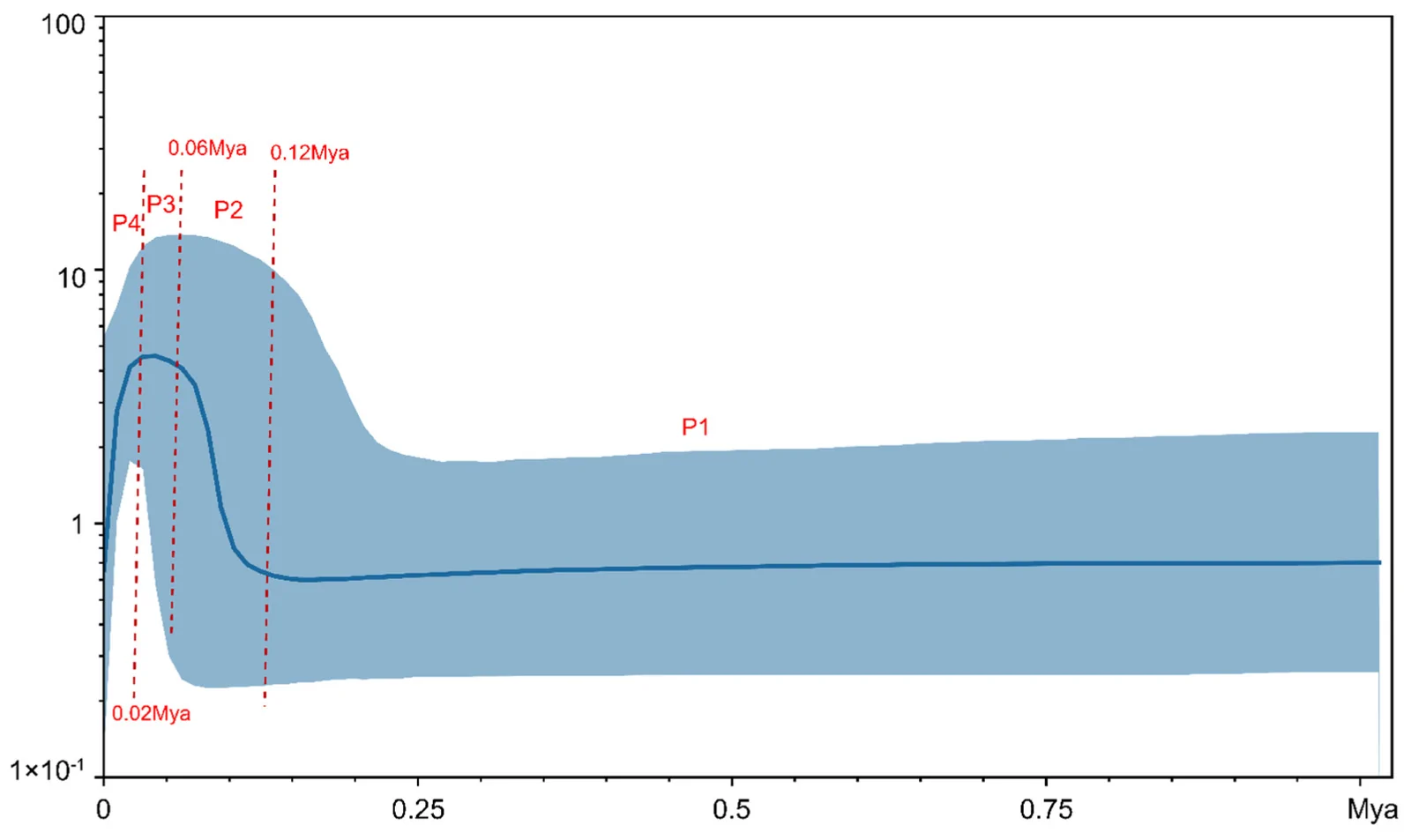
Historical population dynamics of *W. pigra* based on the Bayesian skyline model and mitochondrial genes (Mya denotes million years ago).

The BSP (Figure 5) analysis indicated that *W. pigra* has experienced pronounced demographic fluctuations since the late Middle Pleistocene, broadly corresponding to Quaternary glacial–interglacial cycles. Based on these changes, the demographic history can be divided into four consecutive phases: P1 (~1.0–0.12 Mya) with stable low Ne; P2 (~0.12–0.06 Mya) with expansion peaking around the Last Interglacial (LIG); P3 (~0.06–0.02 Mya) with sharp decline; and P4 (<0.02 Mya) with further rapid decline to a low level, likely exacerbated by anthropogenic impacts. This pattern indicates long-term low-level stability, expansion, rapid decline, and modern contraction. However, the use of an insect mitochondrial rate for annelids introduces uncertainty in timing, so these estimates should be interpreted cautiously.

To further validate these demographic dynamics, we conducted Tajima’s D, Fu’s Fs, and mismatch distribution analyses (Table 6, Figures S7–S10). The coexistence of high haplotype diversity and low nucleotide diversity in most populations typically conforms to a historical bottleneck followed by expansion model. At the species level, the significantly negative Fu’s Fs value under the standard neutral model (Fs = −6.002, *p* = 0.001) indicates an excess of rare mutations, reflecting the genetic imprint left by historical expansion (P2). However, under a population growth model, Tajima’s D (*p* = 0.222), Fu’s Fs (*p* = 0.391), and the Raggedness index (*p* = 0.294) did not deviate significantly from expectations. This suggests that while the overall data do not conflict with an expansion model, the multimodal mismatch distribution further hints at significant internal population substructure. In other words, the overall species-level signal is likely a composite of highly heterogeneous histories across lineages, rather than a single synchronous expansion event.

Lineage-level analyses corroborate this heterogeneity and explain the multimodal distribution observed in the overall data. The Central group exhibited a strong signal of recent expansion: Tajima’s D (*p* = 0.007), Fu’s Fs (*p* = 0.017), and the Raggedness index (*p* = 0.009) were all significant, although its bimodal mismatch distribution suggests localized expansion with substructure. In contrast, the Southern (*p* > 0.10; Fs = 0.760, *p* = 0.864) and Northeastern (*p* > 0.10; Fs = 2.172, *p* = 0.935) groups show non-significant values across all tests, with multimodal mismatch distributions, indicating no recent expansion. Notably, the highly multimodal distribution and positive Fu’s Fs of the Northeastern group strongly point to a long-term independent demographic history characterized by prolonged isolation and genetic drift, rather than recent population growth.

Collectively, the BSP and neutrality tests provide a robust reconstruction of the demographic dynamics of *W. pigra*: the BSP reveals a historical expansion (P2) followed by a recent contraction (P3–P4). The reason neutrality tests still capture the genetic imprint of historical expansion is that the recent contraction has not yet fully erased the old signal—there is a lag in the population reaching a new mutation-drift equilibrium. Therefore, the demographic dynamics of *W. pigra* are the historical product of combined natural climatic oscillations and recent anthropogenic disturbances, exhibiting high lineage specificity rather than synchronous responses. Evidence of long-term independent evolution (prolonged isolation and genetic drift) in the Northeastern (HLJ) group further supports its status as a highly differentiated mitochondrial lineage and a candidate ESU.

## 4. Discussion

In this study, based on concatenated sequences of *COI*, *Cytb*, and *ND1* (3554 bp) from 137 *W. pigra* individuals across 13 geographic populations in China, a total of 441 variable sites were detected. Most populations exhibited a pattern of high haplotype diversity (Hd > 0.5) coupled with low nucleotide diversity (Pi < 0.02), and the species-level concatenated dataset also showed high haplotype diversity (Hd = 0.989). A notable exception was JXJA, which had a concatenated Hd of only 0.200, possibly reflecting a more severe local bottleneck or founder effect. This overall pattern of high Hd and low Pi in most populations is widely interpreted as a typical signal of rapid demographic expansion, whereas the reduced diversity in JXJA suggests a distinct local demographic history. Haplotype networks, phylogenetic trees, and population structure analyses consistently supported the division of the 13 populations into three significantly differentiated genetic groups: the northeastern group (HLJ), the southern group (JXJA, JSTZ, HNYY, HBWH), and the northern-central group (TJ, SDLY, SDZZ, AHBB, HBZX). Among them, the HLJ group exhibited extremely high genetic differentiation from all others (F_ST_ 0.902–0.977). Bayesian Skyline Plot analysis reconstructed four phases of population demographic history, closely matching Late Pleistocene climatic fluctuations, indicating that glacial-interglacial cycles have profoundly affected population size changes in this species.

The pattern of high haplotype diversity (Hd) combined with low nucleotide diversity (Pi) revealed in this study is not unique among medicinal leeches. Lin et al. [67], based on 13 mitochondrial protein-coding genes from seven geographic populations of the Asian buffalo leech (Hirudinaria manillensis) in southern China, similarly found that all populations exhibited high haplotype diversity (>0.5) and low nucleotide diversity (<0.005), and accordingly inferred that the species had experienced historical bottlenecks followed by rapid expansion and mutation accumulation. Notably, that study further revealed that haplotype diversity increased logarithmically with gene length (R^2^ = 0.858, *p* < 0.001), while nucleotide diversity showed a nearly perfect alternating low–high pattern (Z = 2.938, *p* = 0.003), suggesting that the interaction between gene length and evolutionary rate must be fully considered when interpreting mitochondrial genetic diversity parameters, and also providing quantitative support for the necessity of the three–gene combined strategy adopted in our study. Trontelj and Utevsky [68] showed, in their phylogeographic study of three medicinal leech species of the genus Hirudo, that despite wide distributions, mitochondrial diversity was generally low, implying Pleistocene bottlenecks and subsequent rapid recolonization from different glacial refugia following selective sweeps. Popa et al. [69], using mitochondrial *COI* and 12S markers on 133 samples of Hirudo verbana from Romania, further confirmed that this species is currently in a phase of population expansion, with wetland coverage and altitude being the primary ecological variables affecting its distribution. These comparative data indicate that medicinal leeches generally share similar bottleneck-expansion histories, but the phylogeographic fragmentation in *W. pigra* in this study is far more pronounced, with F_ST_ values between HLJ and other populations (0.902–0.977) far exceeding the inter–population differentiation levels observed in Hirudo species. This difference can be attributed to niche differentiation: as a non–blood–feeding leech, *W. pigra* lacks the passive dispersal pathways relying on host movement; its limited active dispersal capacity allows geographic isolation to accumulate deeper phylogeographic sorting.

The formation of the three–group genetic structure can be reasonably explained within the framework of geographic barriers, niche differentiation, and historical climatic events. The HLJ population consistently appeared as a highly homogeneous and independent unit in all BAPS clustering schemes (K = 2 to K = 7), with its differentiation level reaching or exceeding that observed among many leech species. The chromosome-level genome of *W. pigra* published by Liu et al. [70] identified 20 antithrombotic gene families; comparative genomic studies by Zhao et al. [71] on three non-blood-feeding Whitmania species showed that although all three have lost blood–feeding habits, they exhibit significant differences in the retention and evolution of antithrombotic–related genes, implying that different geographic populations may have already accumulated considerable genomic divergence, and the extreme differentiation of the HLJ group may be approaching the threshold of subspecific divergence. The genetic differentiation between the southern and northern–central groups is consistent with the Qinling–Huaihe line acting as a biogeographic barrier in East Asia. However, this study did not directly test this hypothesis, so the barrier explanation remains tentative. Future work with landscape-genetic data would be needed to quantify gene flow across the divide and separate the effects of geographic isolation from environmental factors. Gao et al. [40], based on SNP markers, similarly found that five populations could be traced back to three population sources, qualitatively echoing the three major groups identified in our study. Hao et al. [56] based on *COI* and *Cytb* dual–gene analysis, showed that the Beijing population had the highest genetic diversity, while the Tianjin population had the lowest, with phylogenetic trees showing mixed distribution and no significant population expansion signal detected, largely consistent with our finding that the northern–central group (including TJ) had overall low diversity and lacked recent expansion signals, suggesting that this regional population may have maintained a small effective population size over a long period or experienced genetic homogenization due to aquaculture introductions. Oceguera-Figueroa [72] demonstrated that geographic barriers profoundly shaped speciation patterns in the blood-feeding leech genus Haementeria; the Qinling–Huaihe line, as a dual transition zone of climate and wetland ecosystem types, may have played a similar barrier role.

The four-phase population dynamics revealed by Bayesian Skyline Plot analysis precisely correspond to climatic cycles since the Late Pleistocene. The Last Glacial Maximum (LGM, approximately 26.5–19 ka) led to a substantial contraction of freshwater wetland habitats, causing a sharp reduction in the effective population size (Ne) of *W. pigra* and a genetic bottleneck; post-glacial warming and wetland expansion allowed remnant populations to radiate outward from multiple differentiated refugia. This process elucidates the mechanism underlying the high Hd-low Pi pattern: bottleneck events eliminate many ancient haplotypes, while rapid expansion allows a few surviving lineages to radiate numerous closely related haplotypes over a short period, but insufficient time to accumulate high levels of nucleotide divergence. Lin et al. [67] similarly revealed a five-phase demographic trajectory for Hirudinaria manillensis that closely matched climatic events, further confirming that glacial-interglacial cycles have profoundly affected the effective population sizes of leeches. The extreme genetic uniqueness of the HLJ population in our study suggests that it may have survived the LGM in a northern micro-refugium independent of southern refugia, and after deglaciation, it failed to undergo effective gene flow with southern lineages. The hermaphroditic, cross-fertilizing reproductive mode of *W. pigra* and its strict dependence on wetland habitats further intensified reproductive isolation and genetic drift among refugia, promoting pronounced phylogeographic divergence. Neutrality tests provide additional insights into the genetic signature of past demographic changes, complementing the BSP reconstruction. At the species level, the significantly negative Fu’s Fs under the standard neutral model indicates an excess of rare mutations, which is consistent with a historical expansion. However, under a population growth model, all tests were non-significant and the mismatch distribution was multimodal, suggesting that the overall signal may be a composite of heterogeneous lineage histories rather than a single synchronous expansion. Lineage-level analyses support this view: the Central group shows strong expansion signals, whereas the Southern and Northeastern groups show no evidence of recent expansion, with the Northeastern group exhibiting a positive Fu’s Fs and highly multimodal distribution, consistent with long-term isolation and genetic drift. These results are not necessarily contradictory to the BSP; rather, they may reflect the different temporal scales at which demographic signals are recorded. The BSP may capture the recent contraction (P3–P4), while the neutrality tests may retain the genetic imprint of the historical expansion (P2) that has not yet been erased due to the lag in reaching a new mutation–drift equilibrium. Overall, these results suggest that the demographic dynamics of *W. pigra* are likely lineage-specific rather than synchronous.

Taken together, our study clearly demonstrates that Chinese *W. pigra* populations exhibit a significant bottleneck-expansion genetic footprint, and the populations can be divided into three highly differentiated evolutionary clades—northeastern, southern, and northern-central—with the HLJ population as a highly differentiated mitochondrial lineage and a candidate ESU that should be prioritized for further conservation assessment. The Qinling–Huaihe geographic barrier and historical isolation in Northeast China may have contributed to lineage divergence, while Late Pleistocene glacial–interglacial cycles are the primary temporal drivers of population oscillations. This study not only fills the gap in population genetics research on this species based on combined mitochondrial multi-gene analysis, but also provides a new empirical case for the response paradigm of East Asian aquatic invertebrates to Late Pleistocene climate change, and has fundamental guiding value for genetic assessment, sustainable utilization, and selective breeding of medicinal leech resources. However, this study has certain limitations. First, the sample size per locality (10–12 individuals) is modest, which is a common but noteworthy constraint in mitochondrial population genetics. This limited sampling may introduce stochastic sampling error when estimating within-population diversity and differentiation, particularly for populations with extremely low variation such as JXJA (concatenated Hd = 0.200) and for the inference of a distinct evolutionary lineage such as HLJ. Although multiple independent analyses—including haplotype network, phylogenetic tree, BAPS clustering, and pairwise F_ST_—consistently supported the low diversity of JXJA and the genetic distinctiveness of HLJ, these analyses are based on the same set of samples, and therefore the possibility of sampling artifacts cannot be entirely ruled out. Future studies with larger sample sizes and nuclear markers are needed to validate these findings. Second, analyses were based solely on mitochondrial protein-coding genes, which, as maternal markers, can only reflect maternal lineage history and cannot fully capture the overall population genetic characteristics of the species; future studies should incorporate nuclear markers such as microsatellites and SNPs to validate our conclusions from a biparental inheritance perspective. Third, although our sampling covers the main distribution areas of *W. pigra* in China, sampling density in key geographic transition zones (such as both sides of the Qinling–Huaihe line) remains insufficient, potentially affecting accurate resolution of gene flow patterns among populations; future studies should increase sampling density in transition zones and employ landscape genetic methods to quantify the barrier effects of geographic features on gene flow. Fourth, Bayesian Skyline Plot analysis based on mitochondrial gene mutation rates carries some uncertainty in time estimation; future work could combine paleoclimatic data and multiple independent molecular marker mutation rates for cross-validation. Fifth, this study did not involve morphological, ecological, or functional genomic investigations; future multidisciplinary approaches including morphometrics, ecological niche modeling, and transcriptomics could comprehensively elucidate the adaptive evolutionary mechanisms and conservation genetic needs of this species. Finally, given the special status of the HLJ population as a candidate ESU, future research should focus on its current population status, habitat protection, and genetic resource management, and explore whether it meets the taxonomic criteria for species or subspecies revision, thereby providing a more solid scientific foundation for the conservation and sustainable utilization of *W. pigra*.

## 5. Conclusions

This study elucidated the genetic structure of 13 *W. pigra* populations across China using concatenated mitochondrial COI, Cytb, and ND1 sequences. The species-level dataset and most populations showed high haplotype diversity and low nucleotide diversity, with JXJA as a notable exception. This pattern suggests a history of bottleneck followed by expansion, but neutrality tests and mismatch distribution analyses indicate that the expansion signal is lineage-specific: the Central group shows strong recent expansion, whereas the Southern and Northeastern groups do not. The Northeastern group in particular appears to have experienced long-term isolation rather than recent growth. Haplotype network, phylogenetic, and BAPS clustering analyses consistently supported three differentiated mitochondrial lineages: Northeast, Southern, and Northern–Central. The Northeast (HLJ) population is highly genetically distinct and may represent a highly differentiated mitochondrial lineage and a candidate ESU, pending confirmation with nuclear, morphological, and ecological evidence. BSP analyses suggest demographic dynamics may be broadly associated with Late Pleistocene glacial–interglacial cycles, although the timing should be interpreted cautiously owing to the uncertain substitution rate. These findings provide molecular evidence for the conservation and sustainable use of *W. pigra*, and future work should integrate nuclear markers to further explore its adaptive evolution and conservation strategies.

## Figures and Tables

**Table 1:** Basic sampling information for *Whitmania pigra*.

Locality	City, Province	Longitude	Latitude	Sample Size
JXJA	Jian, Jiangxi	114.99	27.11	10
AHBB	Bengbu, Anhui	117.35	32.93	10
AHCZ	Chuzhou, Anhui	118.30	32.31	10
HBWH	Wuhan, Hubei	114.00	30.00	12
HBZX	Zhongxiang, Hubei	112.07	30.42	12
HLJ	Jiamusi, Heilongjiang	130.32	46.48	10
HNXY	Xinyang, Henan	114.09	32.08	10
HNYY	Yueyang, Hunan	113.05	29.22	10
JSTZ	Taizhou, Jiangsu	119.38	32.01	10
SCYB	Yibin, Sichuan	104.64	28.75	11
SDLY	Linyi, Shandong	118.35	35.05	10
SDZZ	Zaozhuang, Shandong	117.24	34.73	10
TJ	Baodi, Tianjin	117.10	39.10	12

**Table 2:** Genetic Variations of Mitochondrial Protein-Coding Genes in *W. pigra*.

Gene	Length	S	Hn	Hd ± SD	Pi ± SD
*COI*	1534	182	78	0.986 ± 0.003	0.01604 ± 0.00194
*Cytb*	1146	155	64	0.975 ± 0.005	0.01904 ± 0.00184
*ND1*	874	104	53	0.955 ± 0.010	0.01232 ± 0.00176
concatenate	3554	441	86	0.989 ± 0.003	0.01609 ± 0.00185

Note: S: Number of variable sites; Hn: Number of haplotypes; Hd: Haplotype diversity; Pi: Nucleotide diversity.

**Table 3:** Genetic diversity parameters of individual mitochondrial genes (*COI*, *Cytb*, and *ND1*) across 13 populations of *W. pigra*.

Population	S			Hd ± SD			Pi ± SD		
	*COI*	*Cytb*	*ND1*	*COI*	*Cytb*	*ND1*	*COI*	*Cytb*	*ND1*
JXJA	0	1	0	0.000 ± 0.000	0.200 ± 0.154	0.000 ± 0.000	0.00000 ± 0.00000	0.00017 ± 0.00013	0.00000 ± 0.00000
AHBB	32	35	9	0.911 ± 0.077	0.933 ± 0.077	0.667 ± 0.163	0.00510 ± 0.00176	0.00704 ± 0.00318	0.00236 ± 0.00075
AHCZ	35	30	11	0.933 ± 0.077	0.867 ± 0.085	0.800 ± 0.100	0.00887 ± 0.00119	0.01268 ± 0.00154	0.00473 ± 0.00063
HBWH	41	31	18	0.909 ± 0.056	0.909 ± 0.056	0.909 ± 0.056	0.00716 ± 0.00132	0.00645 ± 0.00164	0.00515 ± 0.00075
HBZX	31	24	8	0.848 ± 0.104	0.567 ± 0.154	0.567 ± 0.154	0.00400 ± 0.00179	0.00436 ± 0.00249	0.00225 ± 0.00091
HLJ	14	11	9	0.822 ± 0.097	0.800 ± 0.100	0.844 ± 0.103	0.00281 ± 0.00062	0.00250 ± 0.00062	0.00338 ± 0.00044
HNXY	34	23	15	0.867 ± 0.071	0.911 ± 0.062	0.733 ± 0.120	0.00898 ± 0.00179	0.00840 ± 0.00176	0.00625 ± 0.00153
HNYY	17	11	9	0.911 ± 0.077	0.867 ± 0.085	0.867 ± 0.085	0.00336 ± 0.00069	0.00421 ± 0.00046	0.00450 ± 0.00061
JSTZ	41	32	14	1.000 ± 0.046	0.978 ± 0.054	0.911 ± 0.077	0.00874 ± 0.00107	0.01226 ± 0.00206	0.00509 ± 0.00096
SCYB	22	27	7	0.745 ± 0.098	0.564 ± 0.134	0.564 ± 0.134	0.00687 ± 0.00132	0.00804 ± 0.00233	0.00399 ± 0.00081
SDLY	4	5	7	0.689 ± 0.104	0.778 ± 0.091	0.680 ± 0.104	0.00141 ± 0.00021	0.00161 ± 0.00029	0.00389 ± 0.00053
SDZZ	12	6	8	0.711 ± 0.077	0.822 ± 0.103	0.867 ± 0.085	0.00094 ± 0.00028	0.00203 ± 0.00042	0.00259 ± 0.00048
TJ	12	38	13	0.933 ± 0.078	0.894 ± 0.078	0.939 ± 0.058	0.00217 ± 0.00028	0.00643 ± 0.00217	0.00326 ± 0.00059
Total	182	155	104	0.986 ± 0.003	0.975 ± 0.005	0.955 ± 0.010	0.01604 ± 0.00194	0.01904 ± 0.00184	0.01232 ± 0.00176

**Table 4:** Genetic diversity parameters of concatenated mitochondrial genes across 13 populations of *W. pigra*.

Population	S	Hd ± SD	Pi ± SD
JXJA	1	0.200 ± 0.154	0.00006 ± 0.00004
AHBB	76	0.933 ± 0.092	0.00505 ± 0.00209
AHCZ	76	0.933 ± 0.077	0.00906 ± 0.00110
HBWH	90	0.909 ± 0.056	0.00644 ± 0.00117
HBZX	63	0.848 ± 0.104	0.00369 ± 0.00179
HLJ	34	0.844 ± 0.103	0.00285 ± 0.00053
HNXY	72	0.911 ± 0.062	0.00812 ± 0.00164
HNYY	37	0.911 ± 0.077	0.00391 ± 0.00046
JSTZ	100	1.000 ± 0.045	0.00879 ± 0.00133
SCYB	56	0.745 ± 0.098	0.00768 ± 0.00152
SDLY	16	0.778 ± 0.091	0.00208 ± 0.00027
SDZZ	28	0.978 ± 0.054	0.00216 ± 0.00026
TJ	65	0.939 ± 0.058	0.00493 ± 0.00145
Total	441	0.989 ± 0.003	0.01609 ± 0.00185

**Table 5:** Paired genetic differentiation (F_ST_) of the *W. pigra* population based on mitochondrial genes.

	JXJA	AHBB	AHCZ	HBWH	HBZX	HLJ	HNXY	HNYY	JSTZ	SCYB	SDLY	SDZZ	TJ
JXJA	-	<0.001	<0.001	<0.001	<0.001	<0.001	<0.001	<0.001	<0.001	<0.001	<0.001	<0.001	<0.001
AHBB	0.822	-	0.106	<0.001	0.002	<0.001	0.118	<0.001	<0.001	0.073	<0.001	0.089	0.317
AHCZ	0.609	0.147	-	0.002	0.008	<0.001	0.125	<0.001	0.085	0.290	<0.001	0.013	0.026
HBWH	0.478	0.547	0.289	-	<0.001	<0.001	<0.001	<0.001	0.009	<0.001	<0.001	<0.001	<0.001
HBZX	0.861	0.149	0.291	0.606	-	<0.001	0.011	<0.001	<0.001	0.014	<0.001	<0.001	<0.001
HLJ	0.974	0.932	0.897	0.915	0.944	-	<0.001	<0.001	<0.001	<0.001	<0.001	<0.001	<0.001
HNXY	0.686	0.066	0.067	0.386	0.191	0.906	-	<0.001	0.002	0.124	<0.001	0.002	0.065
HNYY	0.652	0.683	0.448	0.205	0.736	0.941	0.537	-	<0.001	<0.001	<0.001	<0.001	<0.001
JSTZ	0.474	0.459	0.122	0.113	0.550	0.898	0.306	0.274	-	0.007	<0.001	<0.001	<0.001
SCYB	0.682	0.121	0.006	0.398	0.280	0.907	0.089	0.543	0.245	-	0.006	<0.001	0.035
SDLY	0.930	0.201	0.368	0.676	0.376	0.958	0.257	0.801	0.609	0.278	-	<0.001	<0.001
SDZZ	0.927	0.040	0.321	0.669	0.201	0.957	0.194	0.798	0.601	0.280	0.327	-	<0.001
TJ	0.810	0.019	0.201	0.556	0.176	0.932	0.083	0.686	0.485	0.173	0.209	0.128	-

Note: “-” indicates omitted comparisons between identical populations; values below the diagonal represent paired F_ST_ values, while those above the diagonal indicate differentiation *p*-values.

**Table 6:** Demographic parameters and neutrality tests for *W. pigra*.

Dataset	n	Tajima’s D	*p*(D)	Fu’s Fs	*p*(F_S_)	Raggedness r	*p*(r)	Mismatch Shape
**Species-level dataset**	137	−1.061	0.222	−6.002	0.391	0.0020	0.294	Multimodal
**Southern group**	53	−0.894	0.297	0.760	0.864	0.0083	0.501	Multimodal
**Central group**	74	−1.727	0.007	−13.033	0.017	0.0028	0.009	Bimodal
**Northeastern group**	10	−0.757	0.276	2.172	0.935	0.2056	0.925	Highly multimodal

## Data Availability

Data are contained within the article and Supplementary Files.

## Supplementary Materials

The following supporting information can be downloaded at: https://www.mdpi.com/article/10.3390/genes17101278/s1, the assembled mitochondrial gene sequences (*COI*, *Cytb*, and *ND1*) of all 137 individuals have been deposited in the Figshare repository under DOI: 10.6084/m9.figshare.34091109. File S1: all mitochondrial genes of the 137 *W. pigra* samples. Figure S1: Haplotype network of the *W. pigra* population based on mitochondrial gene sequence *COI*. Figure S2: Haplotype network of the *W. pigra* population based on mitochondrial gene sequence *Cytb*. Figure S3: Haplotype network of the *W. pigra* population based on mitochondrial gene sequence *ND1*. Figure S4: Phylogenetic relationships of haplotypes in the *W. pigra* population based on mitochondrial gene sequence *COI*. Figure S5: Phylogenetic relationships of haplotypes in the *W. pigra* population based on mitochondrial gene sequence *Cytb*. Figure S6: Phylogenetic relationships of haplotypes in the *W. pigra* population based on mitochondrial gene sequence *ND1*. Figure S7: Mismatch distribution analysis for the species-level dataset of *W. pigra*. Figure S8: Mismatch distribution analysis for the Southern group of *W. pigra*. Figure S9: Mismatch distribution analysis for the Central group of *W. pigra*. Figure S10. Mismatch distribution analysis for the Northeastern group (HLJ) of *W. pigra*.

## Abbreviations

S	number of variable sites
Hn	number of haplotypes
Hd	haplotype diversity
Pi	nucleotide diversity
