# Supplementary material for: Population Genetics of the Medicinal Leech Whitmania pigra Based on Mitochondrial Genes
Source: Genes (Basel). 2026 Oct 14;17(10):1278. doi: 10.3390/genes17101278 (PMC13704170; doi:10.3390/genes17101278)
Supplement: Supplementary file 1 [file genes-17-01278-s001.zip › Figure S1-S10.pdf]

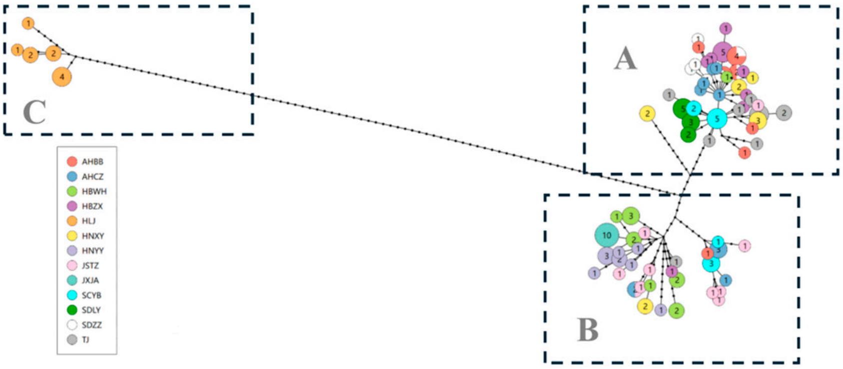

Figure S1. Haplotype network of the *W. pigra* population based on mitochondrial gene sequence *COI.*

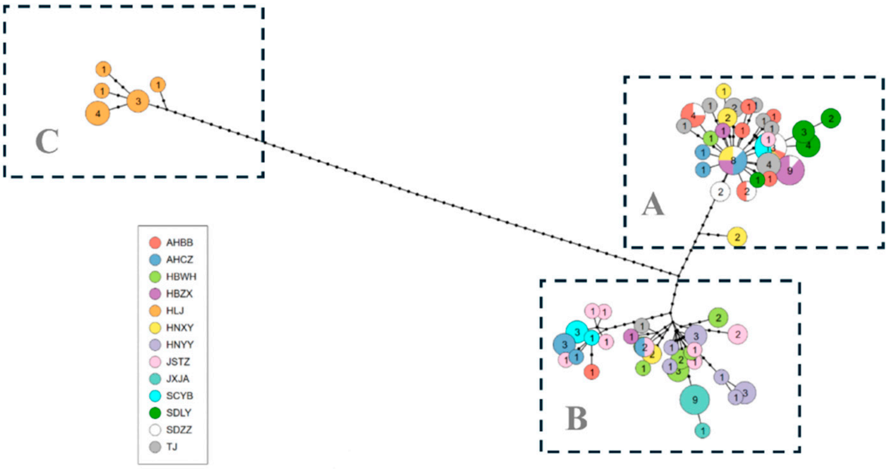

Figure S2. Haplotype network of the *W. pigra* population based on mitochondrial gene sequence *Cytb.*

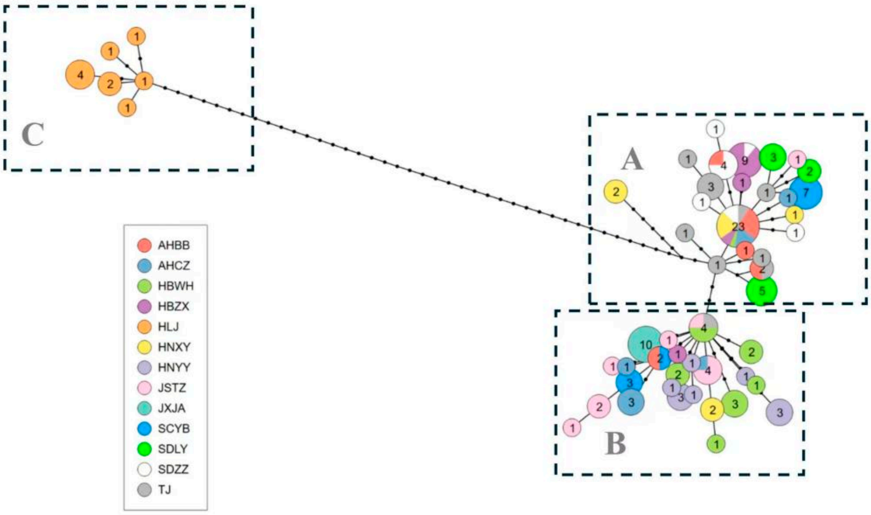

Figure S3. Haplotype network of the *W. pigra* population based on mitochondrial gene sequence *ND1.*

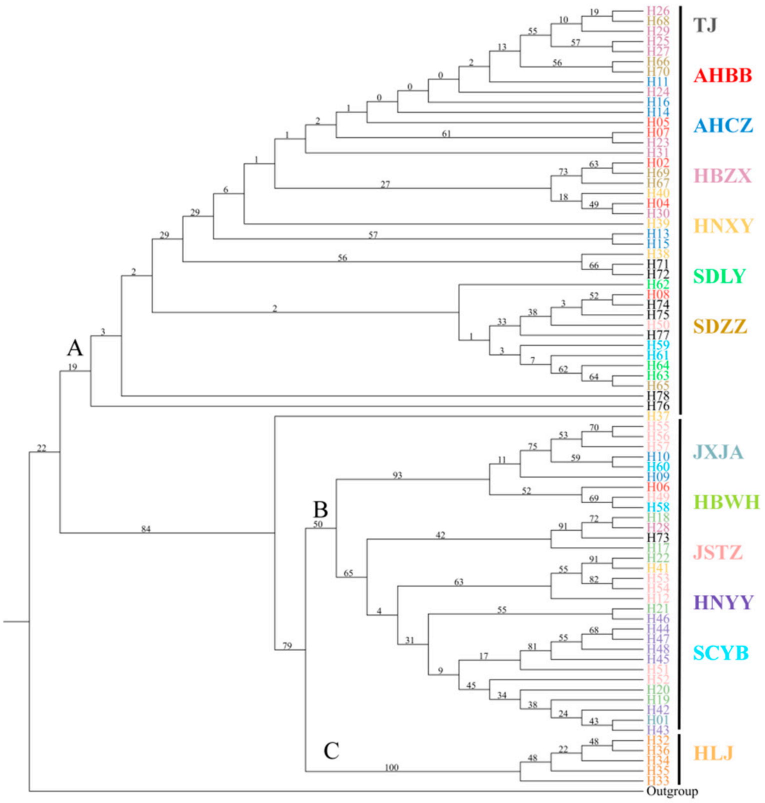

Figure S4. Phylogenetic relationships of haplotypes in the *W. pigra* population based on mitochondrial gene sequence *COI*.

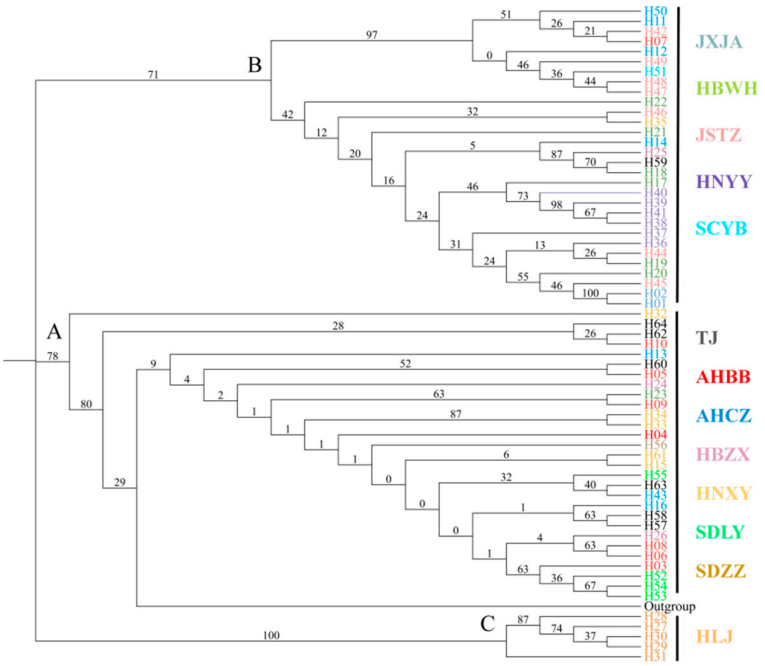

Figure S5. Phylogenetic relationships of haplotypes in the *W. pigra* population based on mitochondrial gene sequence *Cytb*.

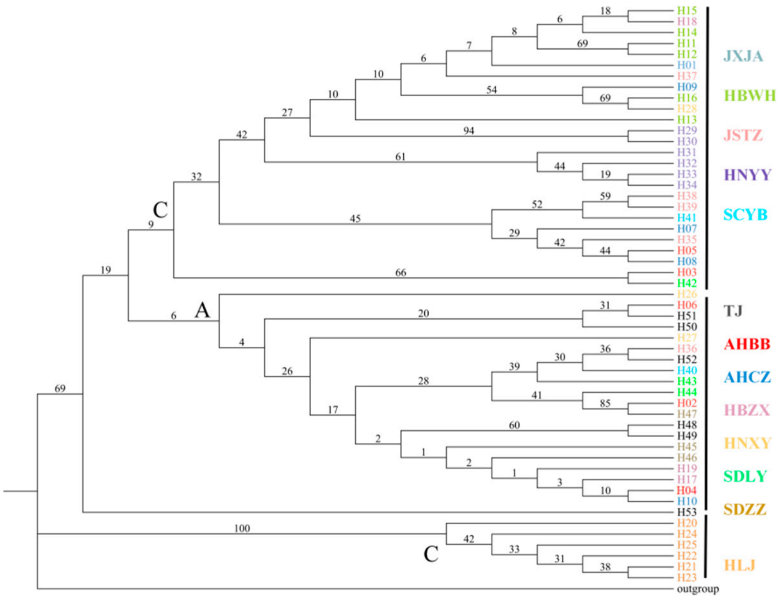

Figure S6. Phylogenetic relationships of haplotypes in the *W. pigra* population based on mitochondrial gene sequence *ND1*.

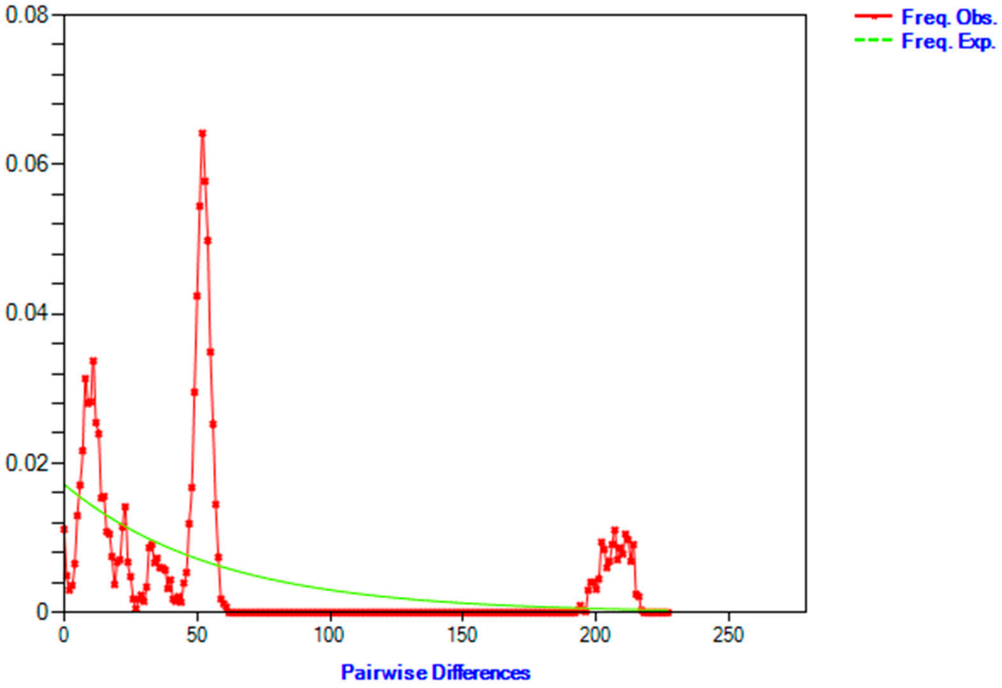

Figure S7. Mismatch distribution analysis for the species-level dataset of *W. pigra*.

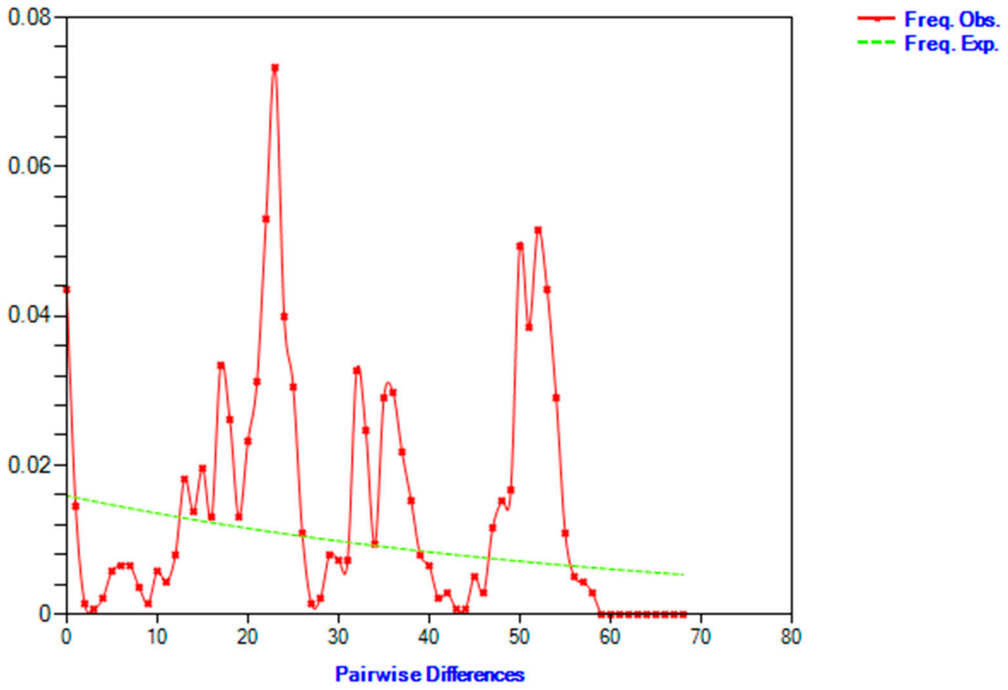

Figure S8. Mismatch distribution analysis for the Southern group of *W. pigra.*

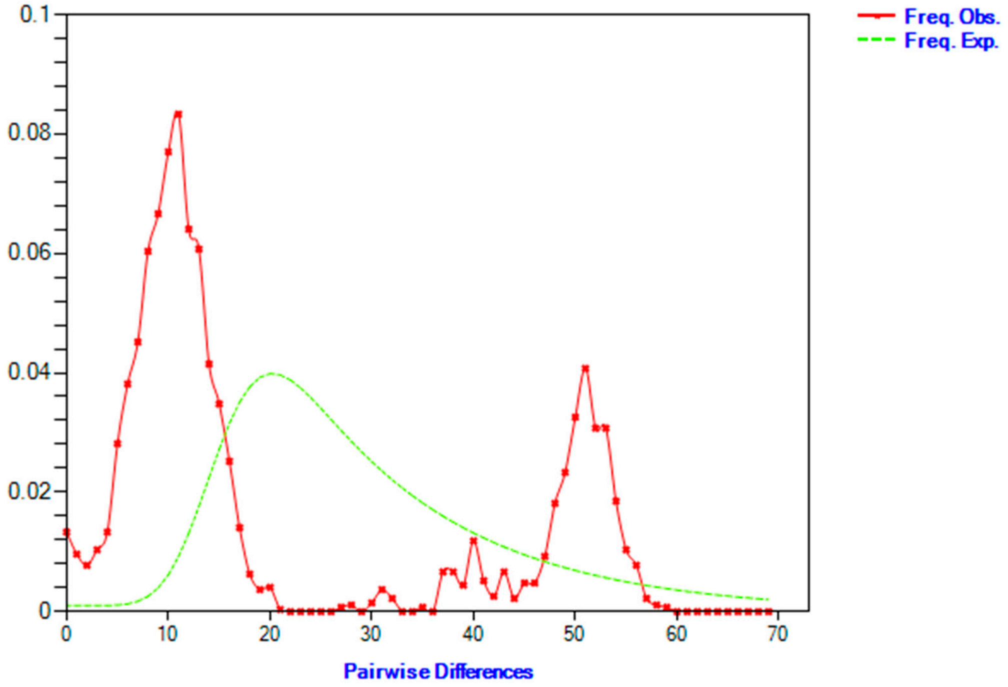

Figure S9. Mismatch distribution analysis for the Central group of *W. pigra.*

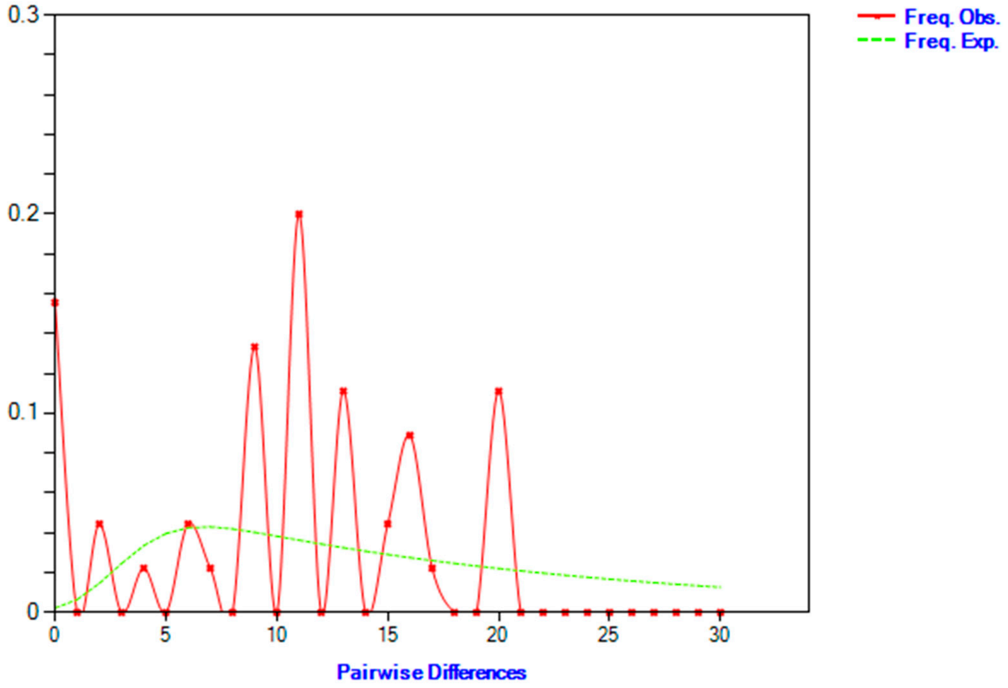

Figure S10. Mismatch distribution analysis for the Northeastern group (HLJ) of *W. pigra*.
